# Multi-cohort transcriptomics integration for building and validating a diagnostic model of peripheral blood septic shock

**DOI:** 10.3389/fimmu.2026.1768866

**Published:** 2026-05-25

**Authors:** Ling Li, Kexun Li, Weiwei Qian, Hui Jiang, Hongqiong Peng, Yang Zhang, Zhengjun Chen, Xia Zeng

**Affiliations:** 1 Emergency Department, Sichuan Provincial People’s Hospital, School of Medicine, University of Electronic Science and Technology of China, Chengdu, China; 2 Emergency Department, Shangjinnanfu Hospital, West China Hospital, Sichuan University, Chengdu, Sichuan, China; 3 Department of Emergency Medicine, Tianjin Medical University General Hospital, Tianjin, China; 4 Robotic Minimally Invasive Surgery Center, Sichuan Provincial People’s Hospital, University of Electronic Science and Technology of China, Chengdu, Sichuan, China

**Keywords:** artificial neural network, diagnostic gene signature, m6a modification, peripheral blood transcriptome, septic shock

## Abstract

**Objective:**

To integrate multi-cohort transcriptomic, single-cell, and experimental data to identify diagnostic signature genes for septic shock, establish a peripheral blood molecular diagnostic model, and elucidate the m6A regulatory mechanisms of key genes.

**Methods:**

Candidate genes were identified from five GEO peripheral blood cohorts through batch effect-corrected differential expression analysis and WGCNA, followed by parallel GO/DO enrichment analysis. Feature genes were selected using PPI networks combined with LASSO, SVM-RFE, and random forest algorithms. A 5-gene artificial neural network (ANN) diagnostic model was constructed and validated using ROC and logistic regression in GSE95233, GSE131761, and clinical cohorts. Immune cell composition and expression of characteristic genes in neutrophils were analyzed using CIBERSORT and GSE167363 single-cell data. The METTL14/YTHDF1–S100A12 m6A axis was elucidated via qRT–PCR, Western blot, MeRIP-qPCR, RIP-qPCR, and Actinomycin D experiments. In CLP mice, siMETTL14 was administered for *in vivo* intervention and assessment of lung injury.

**Results:**

A total of 76 sepsis-shock-associated candidate genes were identified, enriched in the bacterial defense pathway. Five robust candidate genes (S100A12, MMP8, PGLYRP1, CEACAM8, MMP9) were selected by integrating PPI and three machine learning algorithms. The constructed ANN achieved high AUC across multiple cohorts, and all five genes showed significantly elevated mRNA and protein levels in peripheral blood from clinical sepsis patients. CIBERSORT and single-cell results indicated significant neutrophil expansion, with the five genes predominantly enriched in neutrophils and progressively elevated with worsening outcomes. m6A-related experiments demonstrated that METTL14 mediates m6A modification and stabilizes S100A12 mRNA through YTHDF1 recognition; knocking down either METTL14 or YTHDF1 accelerated its degradation. *In vivo* silencing of METTL14 in CLP mice reduced lung tissue injury and lipid peroxidation/ferroptosis levels.

**Conclusion:**

In this study, a diagnostic signature was established for septic shock comprising five neutrophil-associated genes and an ANN model, revealing the regulatory role of the METTL14/YTHDF1-mediated m6A-S100A12 axis in neutrophils. This suggests the METTL14/m6A pathway as a potential diagnostic and therapeutic target.

## 1 Introduction

Sepsis and its progression to septic shock remain one of the most significant causes of death in the ICU, with persistently high mortality rates (1). Although guidelines and supportive therapies have been continuously optimized, existing clinical markers such as lactate, CRP, and PCT remain limited in early diagnosis, risk stratification, and reflecting the essence of immune dysregulation. There is an urgent need for more sensitive, specific molecular biomarkers and diagnostic models that can be detected in peripheral blood (2–4).

In recent years, multi-omics and single-cell studies reveal significant remodeling of peripheral blood immune cell lineages in sepsis, particularly involving neutrophil number and phenotypic abnormalities, which is correlated closely with disease severity and adverse outcomes (5, 6). Among these, sepsis-associated genes such as S100A12 and MMP9 have been repeatedly reported to be elevated in multiple transcriptomic and clinical studies (7–10). They have been identified as potential hub genes and diagnostic/prognostic markers, occupying a central position in septic shock (7, 10–12). Concurrently, RNA epigenetic modifications like m6A have been demonstrated to regulate immune cell function and inflammatory responses. However, the precise mechanisms by which m6A modulates the expression stability of key inflammatory genes within neutrophils and its impact on organ injury remain unclear (13–15).

Under this background, this study integrated multi-cohort peripheral blood transcriptome data. Combining differential analysis, WGCNA, PPI topology, and multiple machine learning methods, we systematically screened robust signature genes for septic shock. An artificial neural network (ANN) diagnostic model was constructed based on five peripheral blood genes, validated through multi-layered testing on public cohorts and clinical samples. Further analysis using CIBERSORT and single-cell transcriptomics confirmed their predominant origin from neutrophils. Through MeRIP, RIP, mRNA stability assays, and CLP mouse models, elucidating the role of the METTL14/YTHDF1-mediated m6A-S100A12 axis in sepsis-associated lung injury. This study aims to provide a novel molecular basis and potential targets for the early diagnosis and targeted intervention of septic shock.

## 2 Materials and methods

### 2.1 Dataset acquisition and preprocessing

Public gene expression datasets were systematically retrieved from the Gene Expression Omnibus (GEO) database using the keywords “septic shock”, “sepsis”, “systemic inflammatory response syndrome”, and “peripheral blood”. Specifically, datasets were included if they met the following criteria: (1) human whole-blood samples with microarray-based gene expression data; (2) clear annotation of septic shock and corresponding comparator groups; (3) publicly available raw or normalized expression matrices; and (4) sufficient sample size for integrative analysis. Datasets were excluded if they were based on non-blood tissues, cell lines, animal models, lacked clear phenotype annotation, contained duplicated samples, or did not include a septic shock subgroup.

Based on these criteria, seven peripheral blood microarray cohorts were included in this study. Among them, GSE13904 (Normal = 18, Septic shock = 67), GSE26378 (Normal = 21, Septic shock = 82), GSE26440 (Normal = 32, Septic shock = 98), GSE4607 (Normal = 15, Septic shock = 42), and GSE8121 (Normal = 15, Septic shock = 30) were used as the training set for model construction. GSE95233 (Normal = 22, Septic shock = 51), and GSE131761 (Normal = 15, Septic shock = 81) served as the external validation cohort. All raw microarray data were downloaded from GEO, undergo background correction, normalization, and log2 transformation, then merged by gene name. For genes with multiple probes, the average probe expression value was used as the final gene expression value.

Single-cell RNA sequencing data were obtained from the peripheral blood dataset GSE167363 (Normal = 2, Sepsis survived = 6, Sepsis non-survived = 4) for subsequent analysis of transcriptional characteristics in peripheral immune cells of sepsis patients at the single-cell level.

### 2.2 Batch effect correction, differential expression, and co-expression analysis

Batch effects across the five training cohorts were corrected using the ComBat algorithm, an empirical Bayes method designed to reduce non-biological variation among batches while preserving biological signals (16). The effectiveness of batch correction was assessed by principal component analysis (PCA). Differentially expressed genes (DEGs) were identified between Septic shock and Normal samples based on the integrated expression matrix, with thresholds of |log2FC| > 1 and false discovery rate (FDR) < 0.05. To further identify gene modules associated with septic shock, weighted gene co-expression network analysis (WGCNA) was performed on the integrated dataset. WGCNA is a systems biology approach that clusters genes with similar expression patterns into co-expression modules and relates these modules to clinical traits (17–20). A soft-thresholding power was selected according to the scale-free topology criterion. Key modules significantly associated with septic shock and showing the highest gene significance (GS) were identified. Intersecting these key module genes with DEGs yielded 76 candidate genes. Gene Ontology (GO) and Disease Ontology (DO) enrichment analyses were then performed on these intersecting genes, with FDR < 0.05 considered statistically significant.

### 2.3 Protein-protein interaction network and machine learning for feature gene selection

A protein-protein interaction (PPI) network comprising the 76 candidate genes was constructed using the STRING database (https://string-db.org/) and visualized in Cytoscape. Hub genes were identified using the cytoHubba plug-in in Cytoscape based on four topological algorithms: Degree, Edge Percolated Component (EPC), Maximal Clique Centrality (MCC), and Maximum Neighborhood Component (MNC) (21, 22). Degree reflects the number of direct interactions of a node, whereas EPC, MCC, and MNC evaluate node importance from different local network-topology perspectives. To further reduce dimensionality and identify robust diagnostic biomarkers, three machine learning algorithms were applied: LASSO logistic regression, which performs variable selection by shrinking regression coefficients (23, 24); support vector machine-recursive feature elimination (SVM-RFE), which iteratively removes features with low classification contribution (25, 26); and random forest, an ensemble learning method that ranks variables according to their importance (27–29). The intersection of these three approaches yielded five robust feature genes (S100A12, MMP8, PGLYRP1, CEACAM8, and MMP9).

### 2.4 Construction and validation of artificial neural network diagnostic model

An artificial neural network (ANN) diagnostic model was constructed in R using the neuralnet package based on the normalized expression values of the five feature genes (30). ANN is a multilayer nonlinear modeling method that can learn complex relationships between input variables and output classes (31–33). The model comprised five input nodes, one hidden layer with 5 neurons, and two output nodes corresponding to the Normal and Septic shock groups. The random seed was fixed at 123, and the maximum number of training steps was set to 1,000,000 (stepmax = 1000000). Other parameters were retained at their default settings, including threshold = 0.01, rep = 1, and random weight initialization. As the optimization algorithm was not manually specified, the model was trained using the default resilient backpropagation algorithm with weight backtracking (rprop+), with the default sum of squared errors as the error function and the logistic function as the activation function. No explicit regularization strategy was used. To minimize over-fitting, a compact architecture based on five pre-selected genes was adopted, and the model was further validated in two independent GEO cohorts. Diagnostic performance was evaluated using receiver operating characteristic (ROC) curves and the area under the curve (AUC), with 95% confidence intervals calculated. In addition, univariate and multivariate logistic regression analyses were performed to construct a forest plot and estimate the odds ratio (OR) and corresponding 95% confidence interval for each gene as an independent diagnostic factor. To further assess the performance and potential clinical utility of the 5-gene diagnostic model, calibration analysis, decision curve analysis (DCA), and nomogram visualization were additionally performed.

### 2.5 Pathway enrichment and immune cell infiltration analysis

Samples were stratified into high- and low-expression groups based on the median expression levels of five characteristic genes. GSEA analysis was performed to identify associated KEGG pathways. Immune cell infiltration analysis was conducted using CIBERSORT, a deconvolution algorithm that estimates the relative proportions of immune cell subsets from bulk gene expression profiles based on a signature matrix (34, 35). Using the integrated peripheral blood expression profiles, the proportions of 22 immune cell subpopulations were inferred and compared between the Normal and Septic shock groups.

### 2.6 Single-cell transcriptome analysis

GSE167363 underwent quality control, normalization, dimensionality reduction, and clustering using the standard Seurat workflow. Major immune cell subpopulations were annotated via classical marker genes (36, 37). UMAP plots were generated to visualize peripheral immune cell composition differences across clinical outcome groups (Healthy control, Sepsis survived, Sepsis non-survived). Single-cell expression patterns of five signature genes were analyzed across immune cell types. Neutrophil populations were isolated, and expression differences of the five characteristic genes were compared across the three groups (Healthy control, Sepsis survived, and Sepsis non-survived).

### 2.7 Transient transfection of siRNAs

Fresh blood was collected from septic shock patients. Within 2 hours, human neutrophils were isolated and purified using the Human Neutrophil Isolation Kit (Solarbio) according to the manufacturer’s instructions. Neutrophil viability was determined by trypan blue exclusion staining, confirming > 98% viability. Neutrophils were then seeded in RPMI 1640 medium (Thermo Fisher Scientific) supplemented with 10% FBS and incubated at 37 C in 5% CO_2_. si-METTL14, si-YTHDF1, and corresponding negative control siRNA were transfected into neutrophils using Lipofectamine 3000 (Invitrogen) according to the manufacturer’s protocol. The sequences of the small interfering RNAs are listed in **Supplementary Table 1**. This study protocol was reviewed and approved by the Ethics Committee for Basic and Clinical Research of Sichuan Medical Academy and Sichuan Provincial People’s Hospital (Approval No. 2025379). All subjects were patients admitted to the emergency department or intensive care unit at Sichuan Provincial People’s Hospital who met diagnostic criteria for septic shock. Before the inclusion, written informed consent had been obtained from the patients themselves or their legal representatives. The study was conducted in accordance with the principles of the Declaration of Helsinki.

### 2.8 METTL14 *In Vivo* therapy

A mouse septic shock model was established using cecal ligation and puncture (CLP). During model establishment, negative control Scr siRNA or METTL14 siRNA was administered *in vivo*. The siRNAs used for *in vivo* delivery were chemically modified with 2’-O-methyl (2’ome) and 5’-cholesterol (5’chol). The siRNA sequences are listed in **Supplementary Table 1**. This animal study protocol was approved by the Medical Ethics Committee of Sichuan Medical Academy and Sichuan Provincial People’s Hospital (Approval No. 2024661).

### 2.9 RNA isolation and RT-qPCR assay

Total RNA was extracted from peripheral blood or isolated neutrophils using TRIzol Reagent (Invitrogen) according to the manufacturer’s instructions. Reverse transcription was performed using the PrimeScript RT Master Mix Kit (TaKaRa) to synthesize cDNA. Real-time quantitative PCR was conducted following TaKaRa’s protocol. Primer sequences for target genes S100A12, MMP8, PGLYRP1, CEACAM8, MMP9, METTL14, and YTHDF1 are listed in **Supplementary Tables 2, 3**. GAPDH served as the internal control gene, and relative mRNA expression levels were calculated using the 2^(-ΔΔCt) method.

### 2.10 Protein extraction and western blot

Total protein was extracted from peripheral blood cells or isolated neutrophils using RIPA lysis buffer (Thermo Scientific). Samples were lysed on ice and centrifuged to collect the supernatant. Protein concentration was determined using the BCA method. Equivalent protein samples were loaded onto a 12% SDS-PAGE gel for electrophoresis and transferred to a PVDF membrane. The membrane was blocked with 5% nonfat dry milk at room temperature for 1 hour. Membranes were incubated overnight at 4 C with the following primary antibodies: S100A12 (ab272713), MMP8 (ab81286), PGLYRP1 (18046-1-AP), CEACAM8 (30875-1-AP), MMP9 (10375-2-AP), METTL14 (ab309096), YTHDF1 (17479-1-AP), and β-ACTIN (#4967) as an internal control. After washing the membrane three times with TBST, the corresponding HRP-labeled secondary antibodies (#7074 and #7076) were added, incubated at room temperature for 2 hours, and then washed the membrane three times with TBST. Signal was developed using ECL chemiluminescent reagent (Thermo Scientific) and captured signal via exposure with an imaging system.

### 2.11 MeRIP-qPCR

Total RNA was extracted from human neutrophils treated with si-NC or si-METTL14, with RNA fragmentation performed as needed. MeRIP experiments were conducted using the Magna MeRIP™ m6A Kit (Merck Millipore) and strictly followed the manufacturer’s protocol. Briefly, total RNA (300 μg) was ethanol precipitated (with 1/10 volume sodium acetate, 100 μg/mL glycine, and 2.5× volume anhydrous ethanol). Each sample was incubated with 50 μL A/G magnetic beads and either 10 μg anti-m6A antibody or isotype control IgG. Subsequently, the MeRIP reaction mixture containing RNase Inhibitor was added to the antibody-magnetic bead complex and incubated at 4 C for 2 hours. After incubation, the enriched RNA was eluted and recovered using the kit’s elution buffer. The eluted product was purified using the RNeasy^®^ MiniElute^®^ Cleanup Kit (QIAGEN). Finally, the immunoprecipitated RNA was reverse transcribed and subjected to qRT-PCR to detect the enrichment level of S100A12 mRNA.

### 2.12 RIP-qPCR

After siRNA-NC or siRNA-METTL14 interference in human neutrophils, RNA immunoprecipitation (RIP) was performed using the Magna RIP™ Kit (Millipore) according to the manufacturer’s instructions. Briefly, cell lysates were prepared using RIP lysis buffer, and supernatants were collected. Magnetic beads were incubated separately with anti-YTHDF1 antibody (#ab220162) or isotype control IgG at room temperature to form antibody-magnetic bead complexes. After approximately 30min incubation, cell supernatants were added to the antibody-coated magnetic beads and incubated overnight at 4 C in RIP immunoprecipitation buffer for immunoprecipitation. Subsequently, Proteinase K was added and incubated at 55 C for 30min to digest proteins. Total RNA was extracted using the chloroform method. Following reverse transcription, qRT-PCR was performed to detect the enrichment level of S100A12 mRNA, thereby evaluating the binding capacity of YTHDF1 to the S100A12 transcript and its changes under si-METTL14 intervention conditions.

### 2.13 mRNA stability assay

Following transfection of human neutrophils with si-METTL14 or si-YTHDF1 and corresponding negative controls, Actinomycin D was added to block new transcription. Cells were harvested at 0, 2, 4, and 6 hours, RNA was extracted, and S100A12 mRNA levels were measured. A degradation curve was plotted using the initial time point as reference, and mRNA half-life was fitted to compare differences in S100A12 mRNA stability among different treatment groups.

### 2.14 Tissue immunofluorescence detection

Mouse septic shock lung tissue was fixed with 4% paraformaldehyde, paraffin-embedded, and sectioned. Apoptotic cells were detected using the CF488 Tunel Cell Apoptosis Detection Kit (Servicebio) according to the manufacturer’s instructions. DAPI counterstaining was performed for nuclei as needed. Separate consecutive sections were subjected to immunofluorescence staining for 4-HNE (GB150073) and GPX4 (GB114327) to reflect lipid peroxidation and ferroptosis-related alterations.

### 2.15 Statistical analysis

All data are expressed as mean ± standard deviation (mean ± SD). Comparisons between two groups were performed using the independent samples t-test, while comparisons among multiple groups were conducted using one-way analysis of variance (ANOVA). Categorical variables were analyzed using the chi-square test or Fisher’s exact test. Correlation analysis employed Spearman’s correlation, selected based on data distribution characteristics. Model diagnostic performance was evaluated using ROC curves and AUC, with 95% confidence intervals calculated when necessary. All statistical analyses were performed using R software and GraphPad Prism software. A two-tailed *P*<0.05 was considered statistically significant.

## 3 Results

### 3.1 Identification of candidate genes associated with septic shock

**Figure 1:**
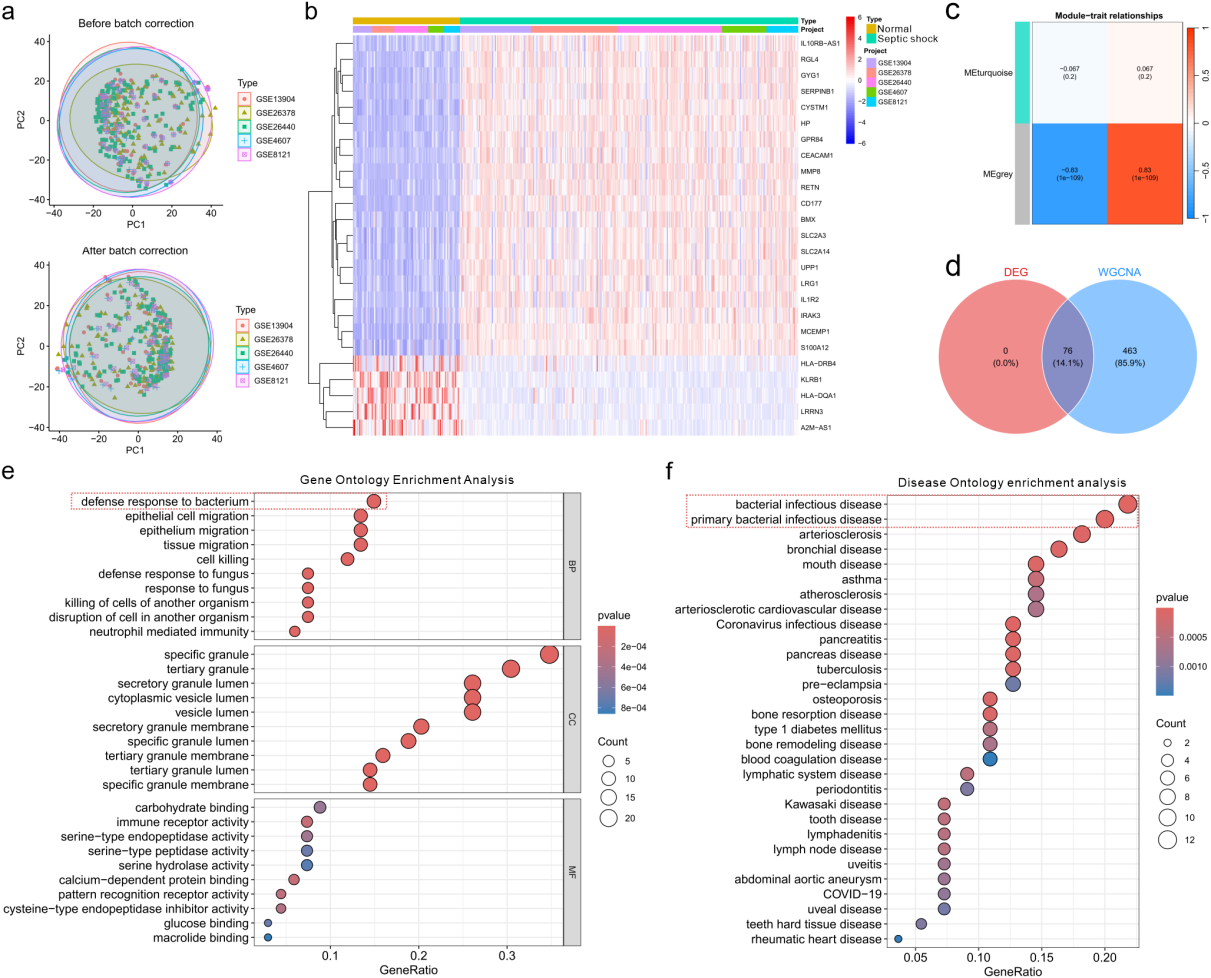
GEO cohort integration and screening of genes associated with sepsis shock. **(a)** Principal Component Analysis (PCA) scatter plots of five microarray expression cohorts (GSE13904, GSE26378, GSE26440, GSE4607, GSE8121) before and after batch effect correction; **(b)** Expression heatmap of DEGs between septic shock patients and healthy controls in the merged peripheral blood cohort; **(c)** Module-trait correlation heatmap of WGCNA-identified module feature genes (MEturquoise, MEgrey) with clinical traits (Normal, Septic shock); **(d)** Venn diagram of DEGs and genes within the grey module; **(e)** GO enrichment analysis results for intersecting genes; and **(f)** Disease Ontology (DO) enrichment analysis results for intersecting genes.

To obtain robust septic shock-associated signature genes, we first integrated five peripheral blood microarray cohorts (GSE13904, GSE26378, GSE26440, GSE4607, GSE8121). Before batch effect correction, PCA revealed sample clustering primarily by cohort origin with batch-specific differences. After correction, samples from different cohorts showed significantly more consistent distribution in PCA space, indicating effective control of cross-cohort technical bias (**Figure 1a**). Subsequently, a set of differentially expressed genes (DEGs) was obtained via comparing transcriptional expression between septic shock and normal peripheral blood samples. Heatmaps revealed distinct expression patterns between the two groups (**Figure 1b**).

Building upon this, a WGCNA co-expression network was constructed based on the integrated expression matrix. No significant outliers were observed in sample clustering. A β value satisfying approximate scale-free characteristics was obtained through soft thresholding for network construction, ultimately yielding multiple co-expression modules (**Supplementary Figures 1a–d**). Module-phenotype correlation analysis revealed that the signature genes of the grey module were significantly associated with the septic shock state, exhibiting markedly higher average gene significance than the turquoise module (**Figures 1c**; **Supplementary Figure 1e**). Consequently, the grey module was identified as the key module most closely related to septic shock.

Through intersecting DEGs and grey module genes, 76 overlapping genes were obtained (**Figure 1d**), and then used as the core candidate gene set for subsequent analyses. GO enrichment analysis indicated these overlapping genes primarily participate in bacterial infection defense (**Figure 1e**). Disease Ontology (DO) analysis further revealed their significant enrichment in disease entries related to bacterial infection (**Figure 1f**). These findings collectively support their potential key role in the development of septic shock from both functional and disease perspectives.

### 3.2 Screening key diagnostic genes for septic shock based on PPI network topology and multiple machine learning algorithms

**Figure 2:**
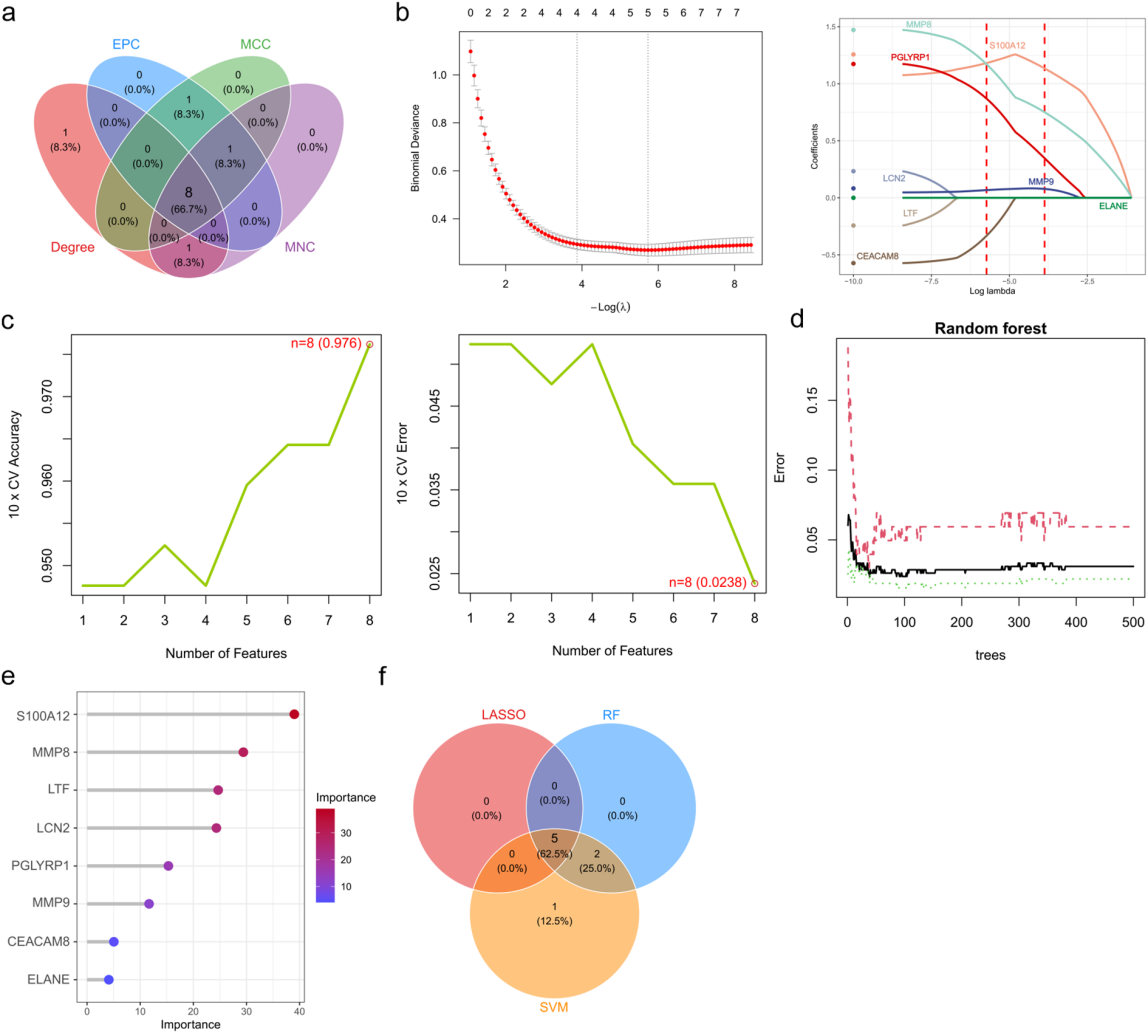
Key diagnostic genes for screening septic shock based on network topology and multiple machine learning algorithms. **(a)** Venn diagram of gene sets screened using four topological algorithms—Degree, EPC, MCC, and MNC—in cytoHubba, constructed from the 76 intersecting genes obtained in **Figure 1** to build a PPI network; **(b)** Results of feature gene selection using the LASSO logistic regression model; **(c)** Results of feature gene selection using Support Vector Machine Recursive Feature Elimination (SVM-RFE); **(d)** Construction results of the random forest classification model; **(e)** Importance ranking diagram of 8 candidate genes in the random forest model; and **(f)** Venn diagram of gene sets selected by three machine learning algorithms: LASSO, random forest, and SVM-RFE.

Building upon the aforementioned 76 intersecting genes associated with septic shock, we first constructed their PPI network using the STRING database (**Supplementary Figure 2**). Using the cytoHubba plugin, we analyzed the network with four topological algorithms—Degree, EPC, MCC, and MNC—to obtain four candidate hub gene sets and further generated a Venn diagram. The results showed that the intersection of the four topological algorithms identified eight highly connected hub genes, considered candidate diagnostic genes with potential key regulatory roles in the network (**Figure 2a**).

To further screen robust diagnostic features from statistical and predictive perspectives, multiple machine learning methods were introduced for feature selection among the candidate genes. The LASSO logistic regression model demonstrated that model bias first decreased and then stabilized under 10-fold cross-validation as the penalty parameter log (λ) varied. Within the optimal λ range and its 1-SE interval, only 8 genes with non-zero coefficients were retained (**Figure 2b**), indicating that these genes significantly contributed to distinguishing Septic shock from Normal. Support Vector Machine Recursive Feature Elimination (SVM-RFE) analysis revealed that classification accuracy and cross-validation error rate achieved optimal balance at 8 features (**Figure 2c**), highly consistent with LASSO results. After constructing a random forest classification model, both the overall out-of-bag (OOB) error rate and the error rates for the Normal and Septic shock groups gradually decreased and stabilized as the number of decision trees increased. This phenomenon suggests reliable classification performance (**Figure 2d**). Gene importance scores revealed that S100A12, MMP8, LTF, and LCN2 contributed significantly to the random forest model (**Figure 2e**).

By integrating the screening results from the three machine learning algorithms and intersecting their feature gene sets, five overlapping robust candidate genes were ultimately identified (**Figure 2f**). These genes serve as core candidates for subsequent development of sepsis shock diagnostic models and functional mechanism studies.

### 3.3 ANN diagnostic model based on five feature genes and multi-cohort validation

**Figure 3:**
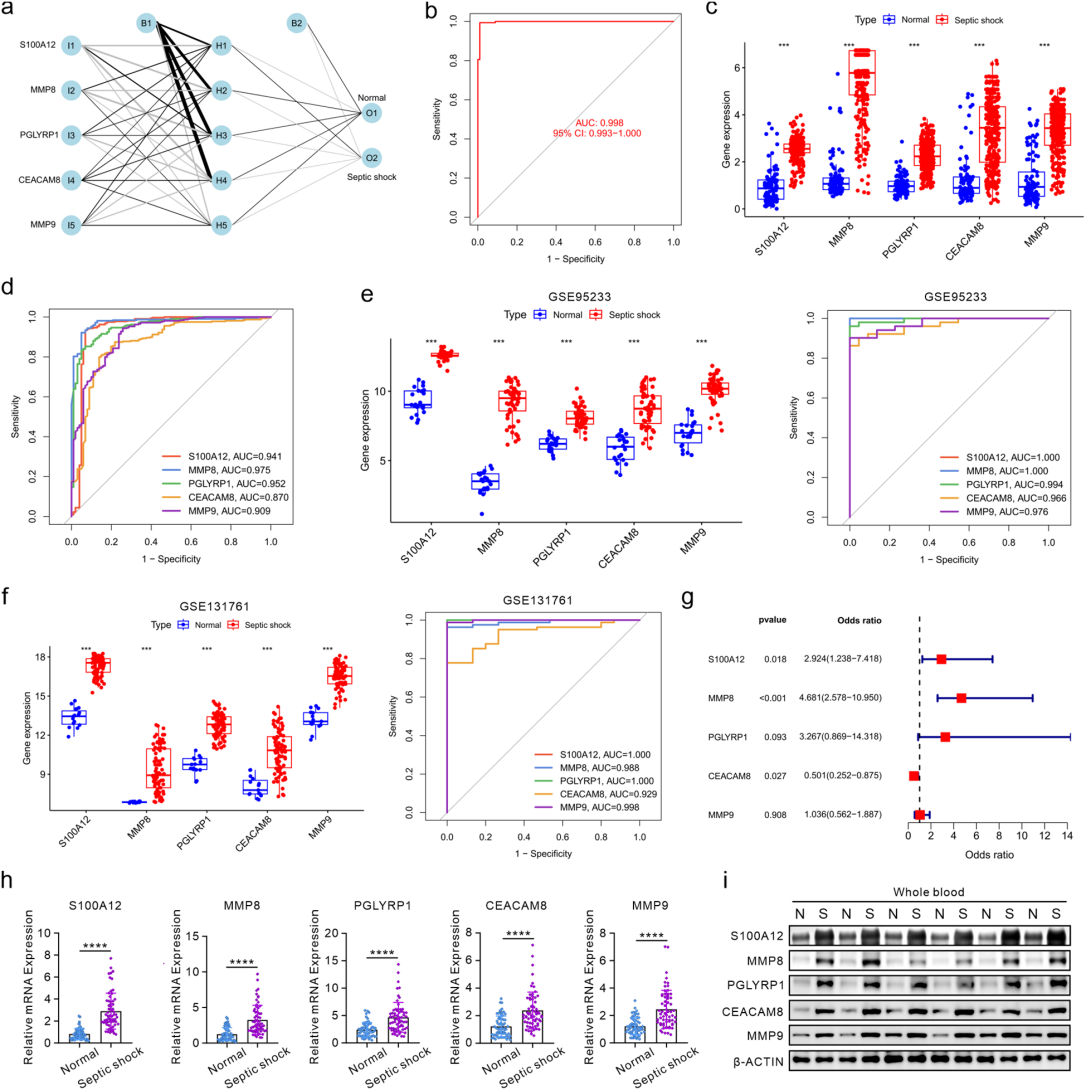
Artificial neural network diagnostic model constructed based on five feature genes and validated across multiple cohorts. **(a)** Schematic diagram of the artificial neural network (ANN) architecture constructed using five feature genes: S100A12, MMP8, PGLYRP1, CEACAM8, and MMP9. The left side shows five input nodes (I1–I5), the middle shows hidden layer nodes (H1–H5), and the right side shows output layer nodes (O1, O2), corresponding to predictions for Normal and Septic shock, respectively. Line thickness indicates weight magnitude; **(b)** Receiver operating characteristic (ROC) curve of the ANN model constructed based on the merged GEO training cohort; **(c)** Box plots showing expression differences of the five feature genes in peripheral blood samples from Normal and Septic shock groups within the merged GEO training cohort; **(d)** ROC curves for each of the five feature genes when used individually to diagnose Septic shock in the merged GEO training cohort. The legend provides the AUC values for each gene, used to evaluate the diagnostic performance of individual genes; **(e)** Box plots of expression differences for the five feature genes in the independent validation cohort GSE95233 (left) and corresponding ROC curves (right); **(f)** Box plots of expression differences for the five feature genes in the independent validation cohort GSE131761 (left) and corresponding ROC curves (right), further validating their stable diagnostic value across different populations; **(g)** Forest plot of the binary logistic regression model constructed based on the five feature genes; **(h)** qRT-PCR validation results of mRNA expression levels for the five feature genes in the clinical peripheral blood cohort. A total of 65 healthy controls (Normal, n = 65) and 65 septic shock patients (Septic shock, n = 65) were included; and **(i)** Western blot results for protein expression of the five characteristic genes in clinical peripheral blood samples. N denotes Normal, S denotes Septic shock. β-ACTIN served as the internal control to validate protein level changes in S100A12, MMP8, PGLYRP1, CEACAM8, and MMP9. ***P < 0.001, ****P < 0.0001.

Furthermore, an ANN diagnostic model was constructed using the expression levels of the five feature genes as inputs (**Figure 3a**). A detailed visualization of the ANN architecture, including the trained connection weights, bias terms, final error, and training steps (**Supplementary Figure 3**). In the combined GEO training cohort, the ROC curve of the ANN model demonstrated a high AUC and narrow confidence interval, indicating its strong discriminatory ability for septic shock (**Figure 3b**). Concurrently, peripheral blood expression levels of all five genes were significantly higher in septic shock patients than in normal controls within the training cohort (**Figure 3c**). Individual gene ROC curves demonstrated diagnostic efficacy for each gene, but overall performance was slightly inferior to that of the combined ANN model (**Figure 3d**). In the independent validation cohorts GSE95233 and GSE131761, the five genes exhibited expression patterns consistent with the training cohort, and their corresponding ROC curves indicated stable diagnostic value in external populations (**Figures 3e, f**). After incorporating all five genes into both univariate and multivariate logistic regression analyses, forest plots revealed that most genes maintained significant odds ratios (ORs) after adjusting for each other’s effects, supporting their validity as independent diagnostic factors for septic shock (**Figures 3g**, **Supplementary Figure 4**). In addition, calibration analysis demonstrated good agreement between predicted and observed probabilities, decision curve analysis indicated a favorable net clinical benefit across a range of threshold probabilities, and the nomogram provided an individualized tool for estimating septic shock risk based on the five-gene signature (**Supplementary Figure 5**).

Finally, in a clinical peripheral blood cohort (Normal n = 65, Septic shock n = 65), qRT-PCR confirmed significant mRNA upregulation of these five genes in the Septic shock group (**Figure 3h**), and Western blot further validated their synchronous elevation at the protein level (**Figure 3i**). Collectively, these five signature genes and the ANN model constructed based on them demonstrated stable and reliable diagnostic performance across multiple cohorts and clinical samples, providing a potential combination of molecular biomarkers for the early identification of septic shock.

### 3.4 Enrichment characteristics of key diagnostic feature gene-related signaling pathways

**Figure 4:**
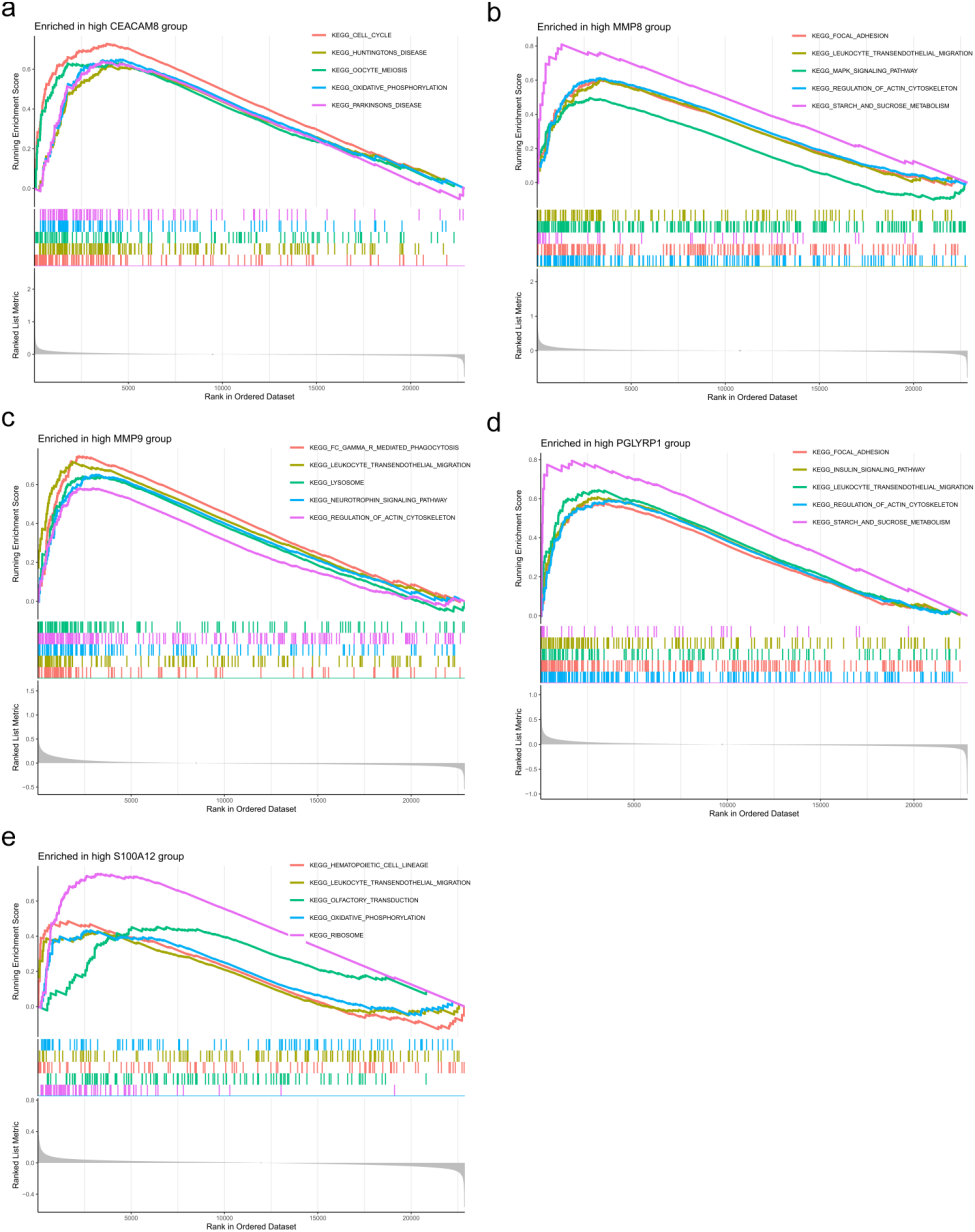
GSEA enrichment analysis of key diagnostic signature genes and associated signaling pathways. **(a)** Gene set enrichment analysis (GSEA) performed on pooled GEO cohort samples stratified by CEACAM8 expression levels into high-expression and low-expression groups; **(b)** GSEA results for MMP8 expression-stratified groups; **(c)** GSEA results grouped by MMP9 expression levels; **(d)** GSEA results grouped by PGLYRP1 expression levels; and **(e)** GSEA results grouped by S100A12 expression levels.

To functionally elucidate the roles of the five feature genes in septic shock, the combined GEO cohort was grouped based on gene expression levels and performed GSEA analysis separately (**Figure 4**). Results showed that in the CEACAM8 high-expression group, pathways including cell cycle, oocyte meiosis, and oxidative phosphorylation were significantly enriched (**Figure 4a**), suggesting its involvement in cell proliferation regulation and energy metabolism reprogramming. In the MMP8 and MMP9 high-expression groups, pathways including focal adhesions, leukocyte transendothelial migration, actin cytoskeleton regulation, phagocytosis, and lysosomes remained enriched (**Figures 4b, c**). This finding supports their involvement in immune cell adhesion, migration, and phagocytic functions. The PGLYRP1-high group showed enrichment in focal adhesions and leukocyte transendothelial migration, alongside upregulation in insulin signaling and starch/sucrose metabolism pathways (**Figure 4d**), suggesting a link between innate immunity and metabolic regulation. In S100A12-high samples, pathways including hematopoietic cell lineages, leukocyte transendothelial migration, oxidative phosphorylation, and ribosomes were significantly enriched (**Figure 4e**), pointing to altered hematopoietic/immune cell composition and enhanced energy metabolism. Collectively, these findings demonstrate that the five signature genes collectively participate in key pathological processes including inflammatory amplification, immune cell recruitment, and metabolic remodeling, providing pathway-level support for their central role in septic shock.

### 3.5 Expression patterns of key characteristic genes at the single-cell level

Further deconvolution analysis of the combined GEO peripheral blood transcriptome using CIBERSORT revealed significant remodeling of immune cell composition between Normal and Septic shock conditions (**Supplementary Figure 6a**). By comparing the infiltration proportions of various cell subpopulations. There was a significant increase in neutrophils in septic shock patients (**Supplementary Figure 6b**), suggesting a potential central role for neutrophils in septic shock.

**Figure 5:**
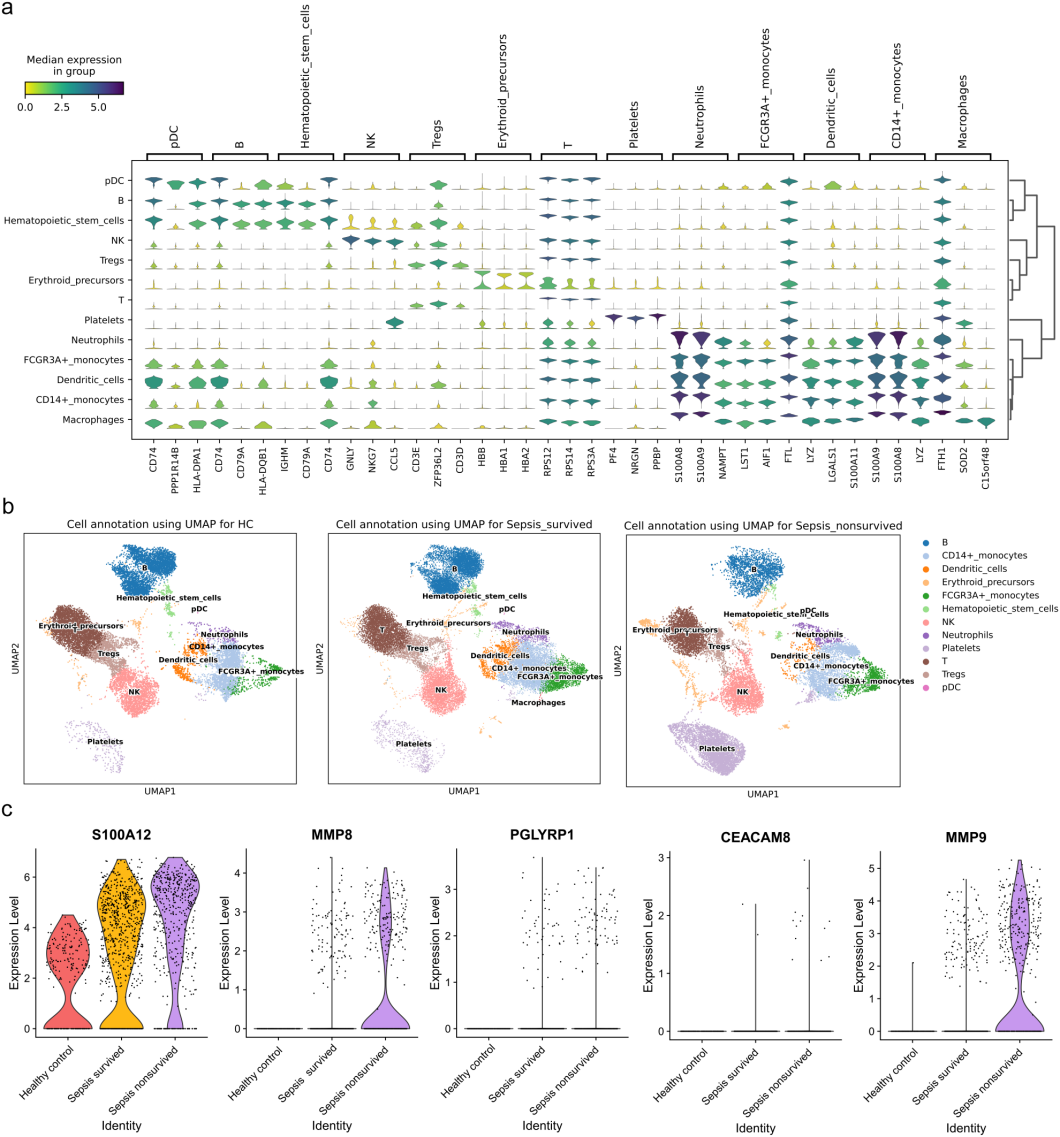
Peripheral immune cell subpopulation segmentation and expression patterns of key characteristic genes at the single-cell transcriptomic level. **(a)** DotPlot of classical immune cell marker genes in peripheral blood single-cell transcriptomic data (GSE167363); **(b)** UMAP visualization generated from all integrated single cells in the single-cell RNA-seq dataset GSE167363, followed by labeling according to disease condition (Healthy control, Sepsis survived, and Sepsis non-survived); **(c)** Violin plots of expression differences for five sepsis shock-associated genes (S100A12, MMP8, PGLYRP1, CEACAM8, and MMP9) in neutrophils across different clinical outcome groups (Healthy control, Sepsis survived, Sepsis non-survived).

Using the single-cell transcriptome dataset GSE167363, we performed detailed annotation of peripheral immune cell subpopulations, identifying multiple cell clusters including neutrophils, monocytes/myeloid cells, lymphocytes, and hematopoietic stem/progenitor cells. Gene expression patterns were clearly labeled, and annotation was reliable (**Figure 5a**). As can be seen from UMAP visualization, there was distinct differences in immune cell composition and distribution among the Healthy control, Sepsis survived, and Sepsis non-survived groups (**Figure 5b**).

Thus, the single-cell expression profiles of five characteristic genes (S100A12, MMP8, PGLYRP1, CEACAM8, MMP9) were analyzed. Therefore, these genes were predominantly enriched in neutrophils, with lower expression in other cell types (**Supplementary Figure 7**). Within neutrophils, expression levels of all five signature genes were significantly higher in both Sepsis survived and Sepsis non-survived groups compared to Healthy control, exhibiting an upward trend with worsening outcomes (**Figure 5c**). This dual confirmation at both immune cell and single-cell levels collectively supports the pivotal role of the “neutrophil-signature gene axis” in sepsis shock development and prognosis.

### 3.6 METTL14/YTHDF1 stabilizes S100A12 mRNA via m6A modification in neutrophils

**Figure 6:**
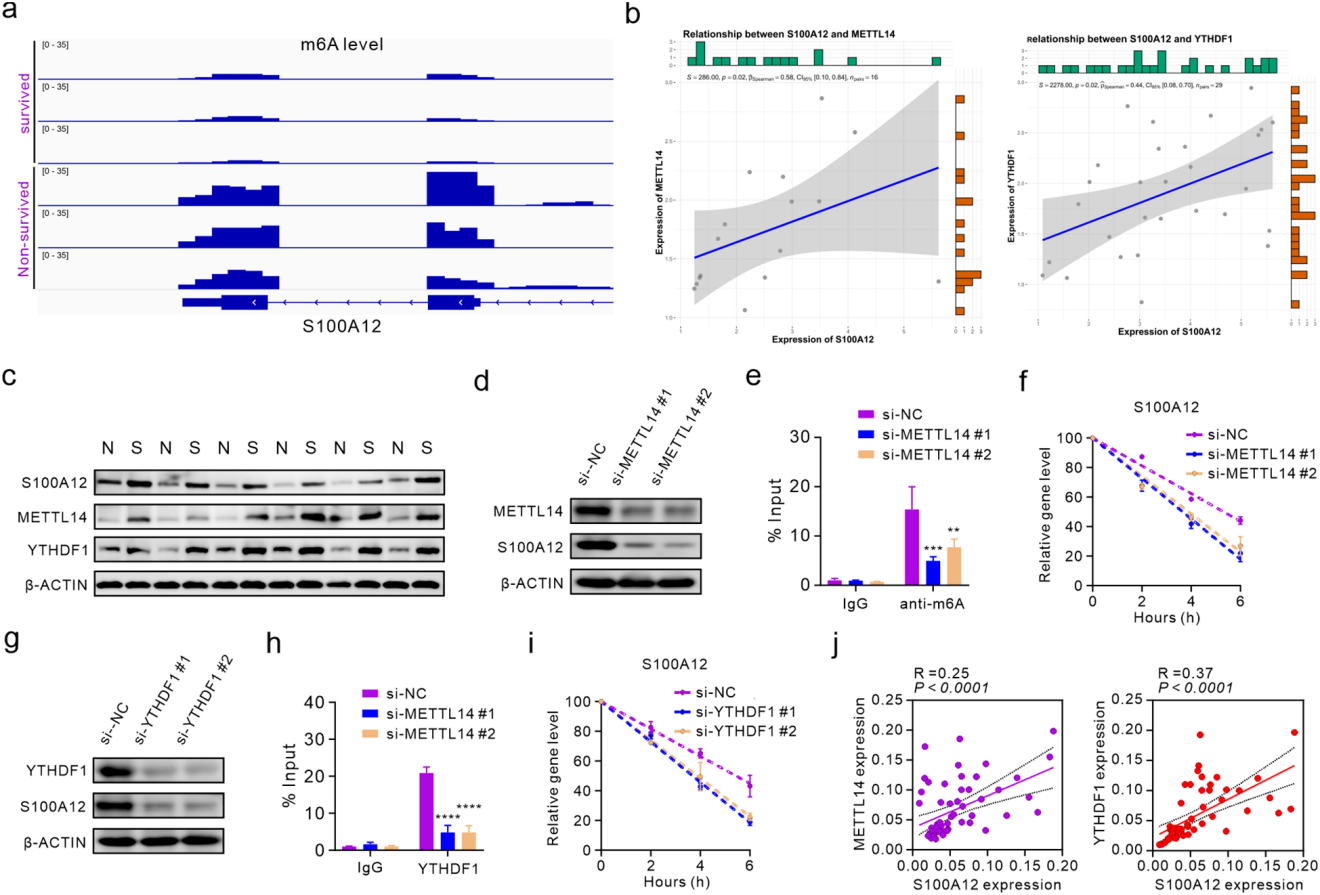
METTL14-mediated m6A modification and YTHDF1 recognition stabilize S100A12 mRNA in neutrophils. **(a)** m6A-seq trace plots reveal m6A modification distribution across S100A12 transcripts in survived versus non-survived groups; **(b)** Single-cell data analysis scatter plots showing correlations between S100A12 and METTL14 (left) and between S100A12 and YTHDF1 (right) mRNA expression in neutrophils; **(c)** Western blot results showing S100A12, METTL14, and YTHDF1 protein expression in peripheral blood-derived neutrophils from normal controls (N) and septic shock patients (S), with β-ACTIN as internal control; **(d)** Detection of METTL14 and S100A12 protein expression in neutrophils cultured *in vitro* after transfection with negative control (si-NC) or two distinct METTL14 siRNAs (si-METTL14 #1, si-METTL14 #2); **(e)** m6A-MeRIP-qPCR was performed in the above neutrophils to detect m6A enrichment on S100A12 mRNA; **(f)** After adding Actinomycin D to neutrophils to block transcription, S100A12 mRNA levels were measured at different time points in si-NC and si-METTL14-treated groups, and degradation curves were plotted; **(g)** Transfected neutrophils with negative control or two distinct YTHDF1 siRNAs (si-YTHDF1 #1, si-YTHDF1 #2) and assessed YTHDF1 and S100A12 protein expression; **(h)** Perform RNA immunoprecipitation (RIP) coupled with qPCR in neutrophils to assess the binding between YTHDF1 and S100A12 mRNA; **(i)** After adding Actinomycin D to neutrophils, detect S100A12 mRNA levels at different time points in si-NC and si-YTHDF1-treated groups and plot degradation curves; and **(j)** Scatter plots showing correlations between S100A12 and METTL14 (left) and between S100A12 and YTHDF1 (right) mRNA expression in peripheral blood cohorts from clinical sepsis shock patients. **P < 0.01, ***P < 0.001, ****P < 0.0001.

In past work, significantly elevated overall m6A levels were found in peripheral blood immune cells from non-survived patients with septic shock. Hence, epigenetic modification differences in key genes across patients with varying prognoses were further analyzed (36). As shown in the m6A-seq analysis, there was more pronounced m6A enrichment peaks on S100A12 transcripts in the non-survived group compared to the survived group (**Figure 6a**), suggesting stronger m6A regulation of S100A12 in patients with poor prognosis. In accordance with single-cell analysis, S100A12 mRNA levels were significantly positively correlated with both METTL14 and YTHDF1 mRNA levels in peripheral blood neutrophils (**Figure 6b**). In clinical samples, S100A12, METTL14, and YTHDF1 proteins were collectively upregulated in peripheral blood-derived neutrophils from septic shock patients (**Figure 6c**).

*In vitro*, METTL14 knockdown in cultured neutrophils markedly reduced S100A12 protein levels (**Figure 6d**) and significantly diminished m6A enrichment on S100A12 mRNA (**Figure 6e**). Actinomycin D tracing experiments further demonstrated that METTL14 deficiency accelerates S100A12 mRNA degradation and shortens its half-life (**Figure 6f**). This implies that METTL14-mediated m6A modification contributes to maintaining S100A12 mRNA stability.

Similarly, YTHDF1 knockdown also resulted in downregulation of S100A12 protein (**Figure 6g**). RIP-qPCR revealed that YTHDF1 specifically enriched S100A12 mRNA, and this binding was significantly weakened upon METTL14 inhibition and reduced m6A levels (**Figure 6h**); Actinomycin D experiments confirmed that YTHDF1 deficiency also accelerates S100A12 mRNA degradation (**Figure 6i**), suggesting its role as an m6A “reader protein” in maintaining transcript stability.

In an independent clinical cohort of peripheral blood from septic shock patients, S100A12 mRNA expression showed significant positive correlations with both METTL14 and YTHDF1 mRNA expression (**Figure 6j**), consistent with the above experimental findings. Collectively, these data demonstrate that in neutrophils, METTL14-mediated m6A modification recognizes and stabilizes S100A12 mRNA via YTHDF1, constituting a key epigenetic transcriptional regulatory axis driving S100A12 overexpression.

### 3.7 METTL14 silencing alleviates sepsis-associated lung injury *in vivo*

**Figure 7:**
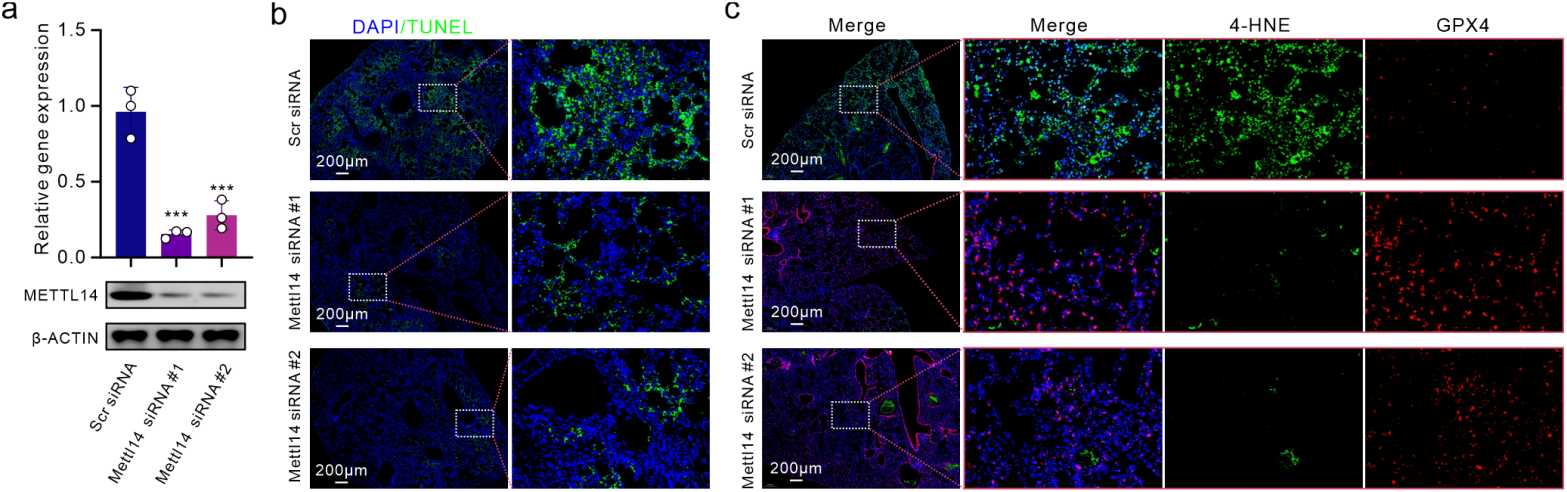
*In vivo* Mettl14 knockdown alleviates pulmonary apoptosis and ferroptosis-related injury in septic mice. **(a)** After establishment of the mouse sepsis model, mice were injected with negative control Scr siRNA or two independent Mettl14 siRNAs (Mettl14 siRNA #1 and Mettl14 siRNA #2), and the knockdown efficiency in lung tissue was evaluated; **(b)** DAPI/TUNEL immunofluorescence staining of lung tissue sections was performed to assess sepsis-associated pulmonary apoptosis. Blue indicates nuclei (DAPI), and green indicates TUNEL-positive apoptotic cells. Scale bar = 200 μm. **(c)** 4-HNE and GPX4 immunofluorescence staining of lung tissue sections was performed to evaluate lipid peroxidation and ferroptosis-related changes. ***P < 0.001.

Given that S100A12 is not expressed in mice, direct intervention targeting S100A12 could not be validated in mouse models (38). Therefore, its upstream key regulatory factor METTL14 was selected as an alternative target for *in vivo* functional validation to assess the impact of this m6A regulatory axis on sepsis-associated lung injury. To validate the functional role of METTL14 in sepsis-associated lung injury, we administered negative control Scr siRNA or two distinct METTL14 siRNA sequences via *in vivo* injection after establishing a mouse sepsis model. Indeed, both METTL14 siRNA sequences significantly downregulated METTL14 mRNA levels in lung tissue compared to the Scr siRNA group, with consistent validation at the protein level via Western blot (**Figure 7a**), indicating reliable knockdown efficiency of METTL14 *in vivo*.

Subsequently, the extent of lung tissue injury was assessed at two levels: apoptosis and lipid peroxidation/ferroptosis. DAPI/TUNEL immunofluorescence staining revealed Scr siRNA-treated sepsis mouse lungs exhibited markedly increased TUNEL-positive cells, whereas METTL14 siRNA #1 and #2-treated groups showed significantly reduced TUNEL-positive apoptotic cells (**Figure 7b**). It can be concluded that METTL14 downregulation mitigates sepsis-induced pulmonary cell apoptosis. Further 4-HNE and GPX4 immunofluorescence staining revealed markedly enhanced 4-HNE-positive signals (a lipid peroxidation marker) and relatively reduced GPX4 expression in Scr siRNA group lungs. Conversely, both METTL14 siRNA groups exhibited significantly attenuated 4-HNE signals and partially restored GPX4 expression (**Figure 7c**). Therefore, *in vivo* silencing of METTL14 reduces pulmonary cell apoptosis and alleviates lipid peroxidation and ferroptosis-related alterations, thereby mitigating overall lung tissue injury in septic mice.

## 4 Discussion

Septic shock remains a major clinical challenge because early identification and precise stratification are often hindered by marked biological heterogeneity (39, 40). In this context, we integrated multi-cohort peripheral blood transcriptomic data and applied a stepwise screening strategy combining differential expression analysis, WGCNA, PPI topology, LASSO, SVM-RFE, and random forest, ultimately identifying five robust feature genes: S100A12, MMP8, PGLYRP1, CEACAM8, and MMP9. Based on these genes, we established a 5-gene ANN model that showed stable diagnostic performance across public datasets and clinical samples, supporting its potential as a peripheral blood molecular diagnostic tool.

Importantly, the value of this 5-gene panel likely extends beyond statistical optimization alone. Although all five genes showed progressively increased expression in neutrophils with worsening disease status, they capture distinct yet functionally connected dimensions of septic shock biology. S100A12 mainly reflects inflammatory amplification and danger-associated signaling (41–43), PGLYRP1 is more closely linked to innate antibacterial defense (44, 45), CEACAM8 represents neutrophil activation and migratory behavior (46), whereas MMP8 and MMP9 are associated with degranulation, extracellular matrix remodeling, endothelial barrier disruption, and tissue injury (47, 48). Therefore, rather than merely providing redundant readouts of neutrophil expansion, this gene combination appears to summarize several key components of a neutrophil-centered pathological network, including inflammatory escalation, host defense, leukocyte activation, and tissue-destructive remodeling. This biological complementarity may help explain why the combined panel outperformed individual markers in diagnostic modeling.

Our immune-cell analyses further support this interpretation. Both CIBERSORT and single-cell transcriptomics consistently showed marked neutrophil expansion and remodeling in septic shock, with the five signature genes displaying progressive upregulation in neutrophils as disease severity worsened. Within this framework, S100A12 is of particular interest as a prototypical neutrophil-derived DAMP closely associated with inflammatory amplification and adverse outcomes (41, 49, 50). Mechanistically, our *in vitro* experiments in human neutrophils showed that METTL14-mediated m6A modification stabilizes S100A12 mRNA through YTHDF1, thereby sustaining its high expression. These findings support the presence of a METTL14/YTHDF1–S100A12 regulatory axis in neutrophils and suggest that m6A-dependent post-transcriptional regulation contributes to the exaggerated inflammatory state of septic shock.

We used a CLP mouse model with systemic administration of METTL14 siRNA to extend these findings *in vivo*. METTL14 silencing alleviated pulmonary apoptosis, lipid peroxidation, and ferroptosis-related injury, indicating that suppression of METTL14-dependent m6A regulation can mitigate sepsis-associated lung injury. However, these *in vivo* results should be interpreted cautiously. Because S100A12 is not expressed in mice, direct interrogation of the human S100A12 pathway was not feasible (38). In addition, intravenously delivered siMETTL14 produced systemic rather than neutrophil-specific knockdown. Therefore, the protective phenotype observed in lung tissue cannot be directly attributed to neutrophil-intrinsic regulation of S100A12 or regarded as definitive proof of a neutrophil-specific METTL14/YTHDF1–S100A12 mechanism. Instead, our animal data are better interpreted as supportive evidence that METTL14-dependent m6A regulation contributes more broadly to sepsis-associated lung injury. This interpretation is also consistent with accumulating evidence that METTL14 participates in multiple sepsis-related pathological programs, including pyroptosis, ferroptosis, oxidative stress, and endothelial dysfunction, in a context-dependent manner (51–58).

Several limitations should therefore be acknowledged. First, although the included GEO cohorts were selected using predefined criteria and externally validated, they may not fully capture the clinical heterogeneity of septic shock, as most discovery datasets were based on public peripheral blood microarray studies and were largely enriched for pediatric populations, whereas broader adult, multicenter, and real-world cohorts remain underrepresented. Second, despite batch correction and independent external validation, residual inter-platform and inter-center heterogeneity may still affect model generalizability. Third, the single-cell dataset was relatively small, and larger cohorts are needed to further validate the immune landscape associated with septic shock. Fourth, our mechanistic work primarily focused on the METTL14/YTHDF1 axis and did not systematically examine other m6A regulators or broader post-transcriptional networks.

The clinical applicability of the diagnostic model also requires further validation. The current ANN model was developed and validated using septic shock and normal control cohorts only, without inclusion of a non-infectious SIRS comparator group. Accordingly, although the 5-gene signature showed robust performance in distinguishing septic shock from healthy controls, its specificity for differentiating infection-related shock from sterile systemic inflammation remains uncertain. In addition, we did not perform a direct head-to-head comparison between the 5-gene ANN model and conventional clinical biomarkers such as PCT, CRP, and lactate, because these data were not consistently available across the included cohorts. Thus, although the proposed transcriptomic signature may reflect a distinct neutrophil-centered immune dysregulation program, its relative or additive diagnostic value over routine clinical markers remains to be determined. Likewise, while the limited size of the 5-gene panel may favor future development as a targeted multiplex assay, its turnaround time, workflow compatibility, and cost-effectiveness in acute ICU/ED settings were not formally assessed in the present study.

Overall, this study establishes a multi-layered framework linking peripheral blood transcriptomics, immune-cell composition, single-cell analysis, and m6A-dependent post-transcriptional regulation in septic shock. We identified a biologically complementary 5-gene diagnostic signature centered on neutrophil dysfunction and provided mechanistic evidence that the METTL14/YTHDF1–S100A12 axis contributes to sustained inflammatory activation in neutrophils and is associated with sepsis-related lung injury. Future studies using neutrophil-specific genetic models, such as Ly6G-Cre- or Mrp8-Cre-mediated conditional deletion of Mettl14, will be required to establish direct causality *in vivo*. At the same time, the 5-gene ANN model should be further optimized and validated in larger and clinically more diverse cohorts, particularly those including non-infectious SIRS controls, and prospective studies will be needed to evaluate its comparative performance against conventional biomarkers as well as the implementation feasibility of a 5-gene multiplex assay in real-world critical care settings.

## 5 Conclusions

This study established a neutrophil-dominant molecular diagnostic signature for septic shock comprising S100A12, MMP8, PGLYRP1, CEACAM8, and MMP9, and developed an ANN diagnostic model supported by multi-cohort validation. Within neutrophils, METTL14-mediated m6A modification recognizes and stabilizes S100A12 mRNA via YTHDF1, forming a critical epigenetic regulatory axis driving S100A12 overexpression and lung injury. This suggests the METTL14/m6A pathway may serve as a potential therapeutic target for sepsis diagnosis and intervention.

## Data Availability

The raw data supporting the conclusions of this article will be made available by the authors, without undue reservation.

## Supplementary material

Supplementary Figure 1 Sample clustering, soft threshold selection, and key module identification based on WGCNA. **(a)** Sample clustering dendrogram for detecting outliers in the merged GEO peripheral blood expression matrix. Each branch represents a sample, used to detect and exclude potential outliers; **(b)** Sample clustering dendrogram and trait heatmap; **(c)** Soft threshold power (β) selection; **(d)** Gene clustering dendrogram and module color distribution; and **(e)** Bar chart of average gene significance (GS) across different modules.

Supplementary Figure 2 PPI network of 76 overlapping genes. A PPI network was constructed using the STRING database, comprising 76 overlapping genes associated with septic shock.

Supplementary Figure 3 Architecture and trained weights of the 5-gene artificial neural network (ANN) diagnostic model for septic shock. The model includes five input neurons, one hidden layer with five hidden neurons, and two output neurons representing the Normal and Septic shock classes. Numbers on the edges indicate the trained connection weights, while blue connections denote bias weights. The overall model error and training steps are shown at the bottom. This ANN model was developed to assess the diagnostic value of the five-gene signature in septic shock.

Supplementary Figure 4 Forest plot of the univariate logistic regression model constructed using five marker genes. The x-axis represents the odds ratio (OR), while the y-axis displays the gene names. The horizontal lines and boxes on the right indicate the OR and its 95% confidence interval, with corresponding p-values annotated on the left. This plot evaluates the contribution of each gene as an independent diagnostic factor for septic shock.

Supplementary Figure 5 Supplementary evaluation and nomogram visualization of the 5-gene septic shock diagnostic model. **(A)** Calibration plot showing the concordance between predicted and actual probabilities for the 5-gene diagnostic model. **(B)** Decision curve analysis demonstrating the clinical net benefit of the model across a range of threshold probabilities. **(C)** Nomogram based on the five feature genes (S100A12, MMP8, PGLYRP1, CEACAM8, and MMP9) for individualized prediction of septic shock risk.

Supplementary Figure 6 Differences in CIBERSORT-estimated peripheral blood immune cell infiltration distribution between Normal and Septic shock. **(a)** Stacked bar chart showing relative proportions of 22 immune cell subpopulations derived from deconvoluted analysis of pooled GEO cohort peripheral blood transcriptome data using the CIBERSORT algorithm; and **(b)** Box plots depicting infiltration ratios of 22 immune cell subpopulations between Normal and Septic shock conditions.

Supplementary Figure 7 Expression patterns of five characteristic genes in peripheral blood monocyte transcriptomes across major immune cell subpopulations and different outcome groups. **(a–c)** DotPlot displays the expression distribution of S100A12, MMP8, PGLYRP1, CEACAM8, and MMP9 across immune cell subpopulations, corresponding to **(a)** Healthy control, **(b)** Sepsis survived, and **(c)** Sepsis non-survived cohorts.
