# Supplementary figures and images for: Multi-cohort transcriptomics integration for building and validating a diagnostic model of peripheral blood septic shock
Source: Front Immunol. 2026 May 25;17:1768866. doi: 10.3389/fimmu.2026.1768866 (PMC13243033; doi:10.3389/fimmu.2026.1768866)

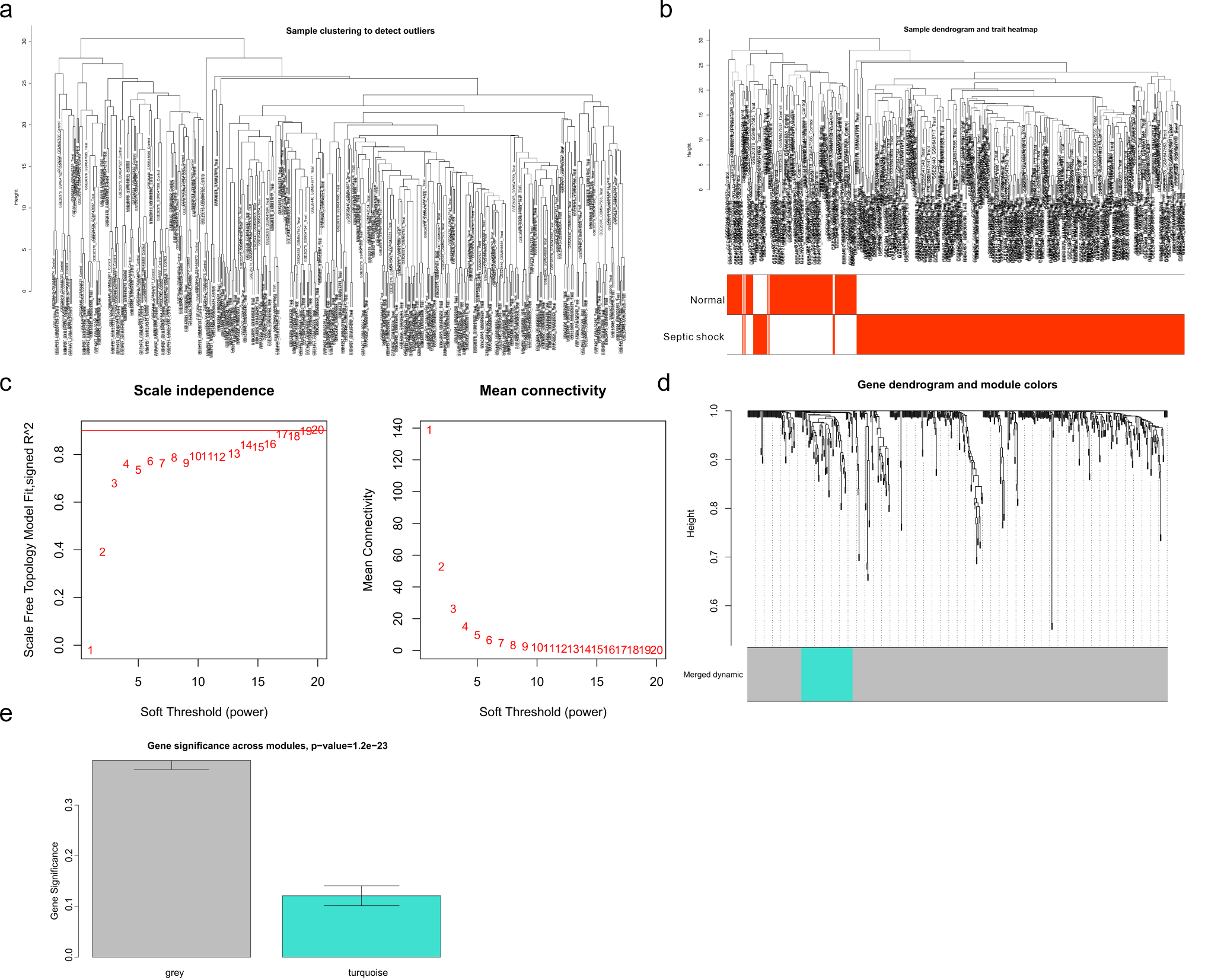

Supplement: Supplementary Figure 1 — Sample clustering, soft threshold selection, and key module identification based on WGCNA. (a) Sample clustering dendrogram for detecting outliers in the merged GEO peripheral blood expression matrix. Each branch represents a sample, used to detect and exclude potential outliers; (b) Sample clustering dendrogram and trait heatmap; (c) Soft threshold power (β) selection; (d) Gene clustering dendrogram and module color distribution; and (e) Bar chart of average gene significance (GS) across different modules. [file Image1.tif]

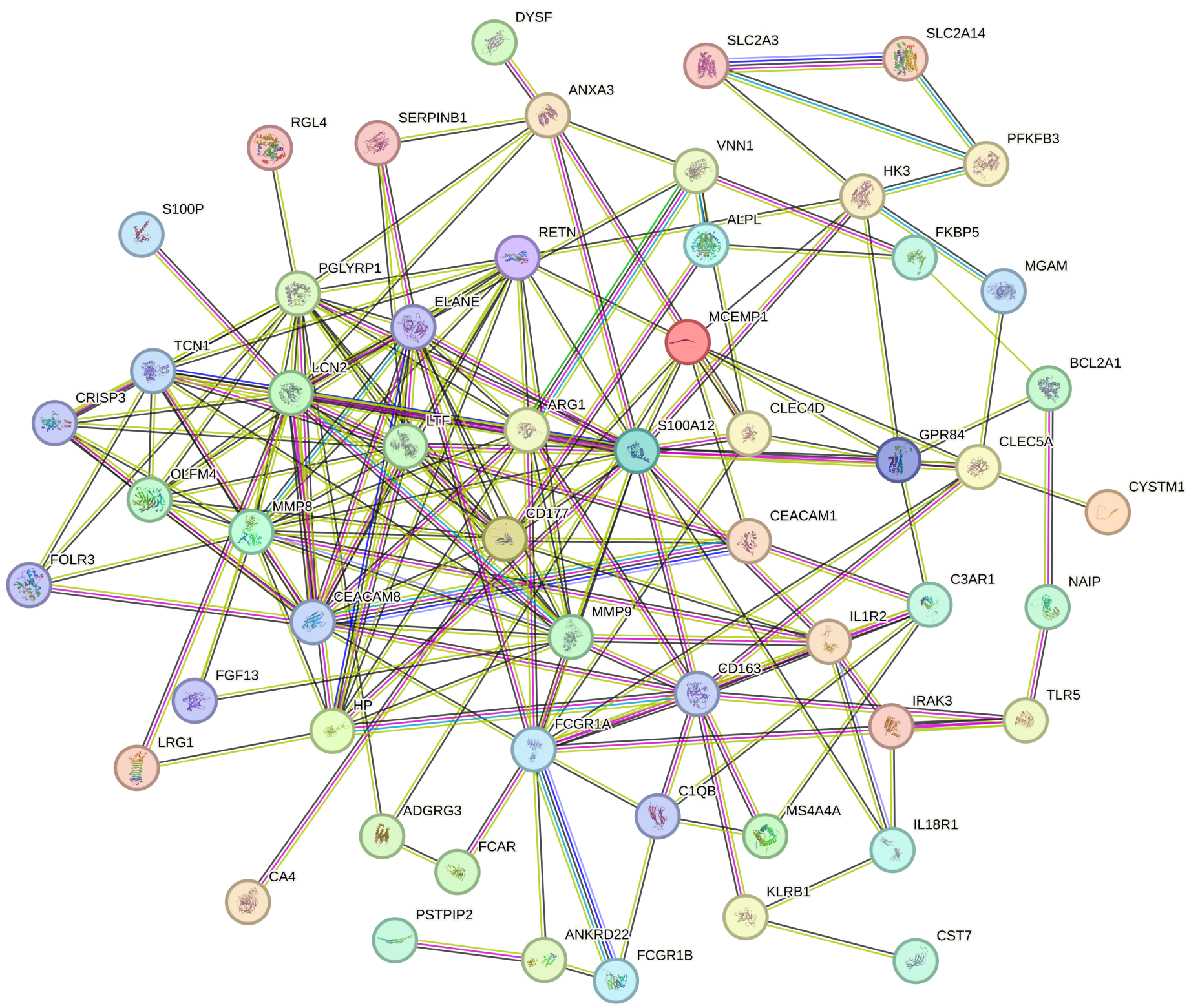

Supplement: Supplementary Figure 2 — PPI network of 76 overlapping genes. A PPI network was constructed using the STRING database, comprising 76 overlapping genes associated with septic shock. [file Image2.tif]

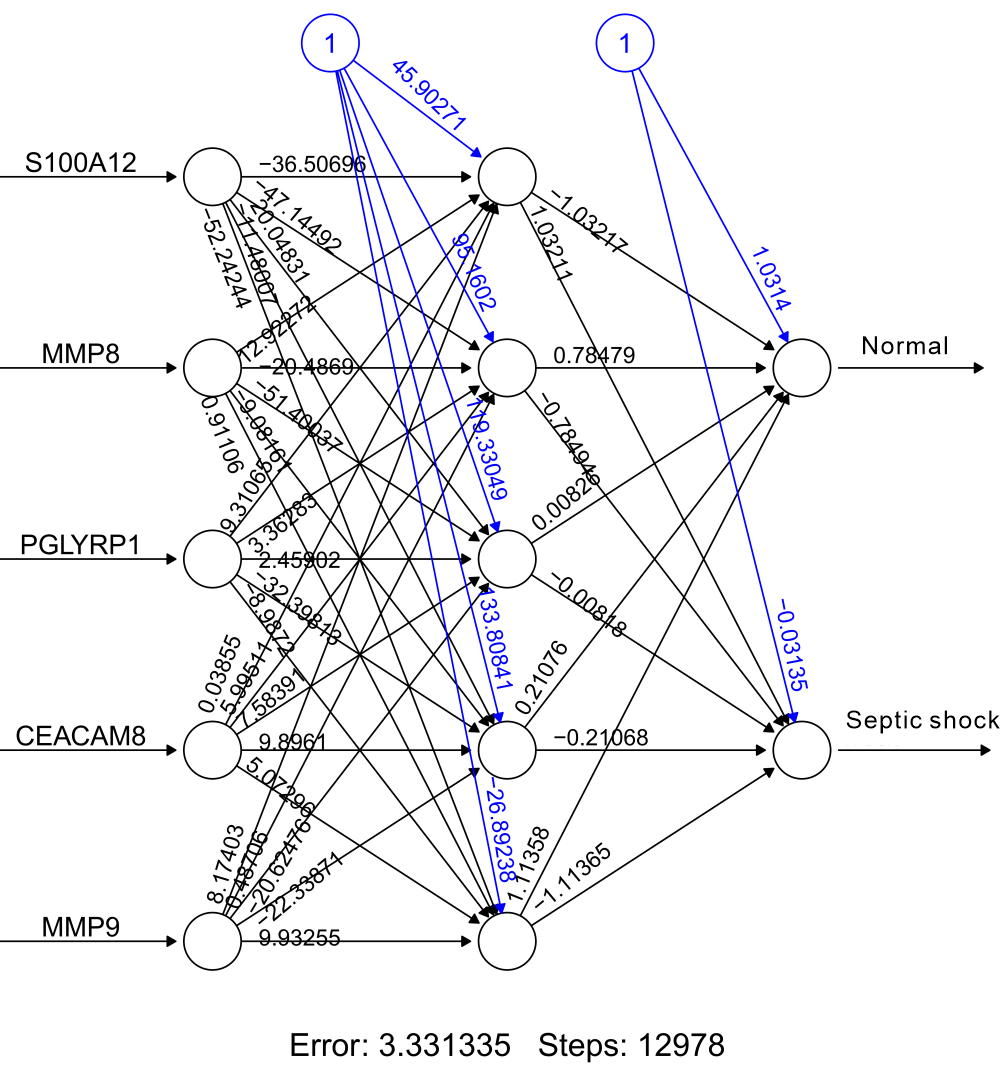

Supplement: Supplementary Figure 3 — Architecture and trained weights of the 5-gene artificial neural network (ANN) diagnostic model for septic shock. The model includes five input neurons, one hidden layer with five hidden neurons, and two output neurons representing the Normal and Septic shock classes. Numbers on the edges indicate the trained connection weights, while blue connections denote bias weights. The overall model error and training steps are shown at the bottom. This ANN model was developed to assess the diagnostic value of the five-gene signature in septic shock. [file Image3.tif]

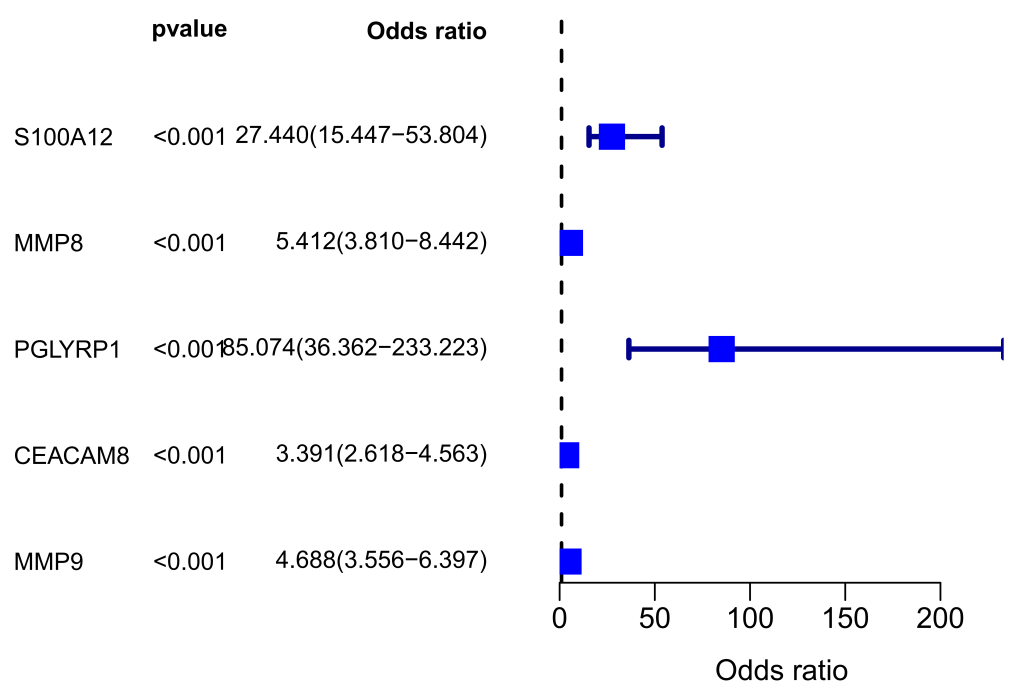

Supplement: Supplementary Figure 4 — Forest plot of the univariate logistic regression model constructed using five marker genes. The x-axis represents the odds ratio (OR), while the y-axis displays the gene names. The horizontal lines and boxes on the right indicate the OR and its 95% confidence interval, with corresponding p-values annotated on the left. This plot evaluates the contribution of each gene as an independent diagnostic factor for septic shock. [file Image4.tif]

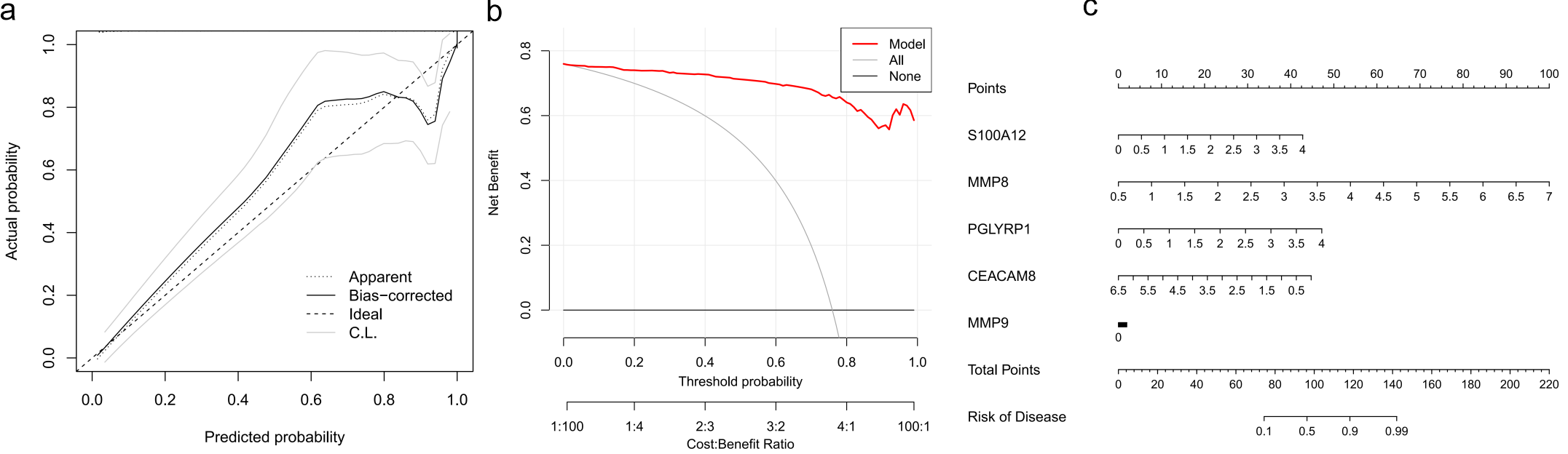

Supplement: Supplementary Figure 5 — Supplementary evaluation and nomogram visualization of the 5-gene septic shock diagnostic model. (A) Calibration plot showing the concordance between predicted and actual probabilities for the 5-gene diagnostic model. (B) Decision curve analysis demonstrating the clinical net benefit of the model across a range of threshold probabilities. (C) Nomogram based on the five feature genes (S100A12, MMP8, PGLYRP1, CEACAM8, and MMP9) for individualized prediction of septic shock risk. [file Image5.tif]

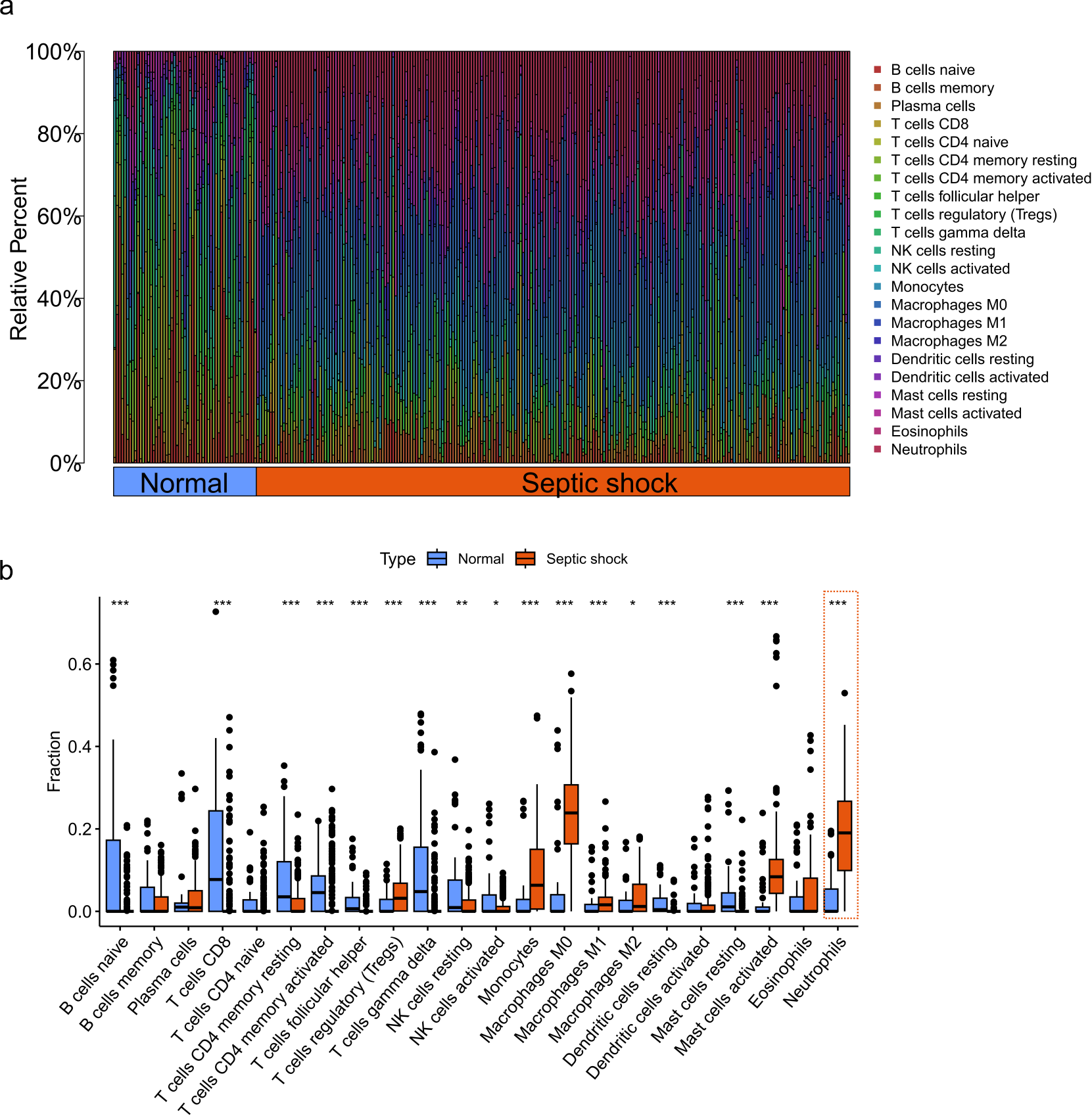

Supplement: Supplementary Figure 6 — Differences in CIBERSORT-estimated peripheral blood immune cell infiltration distribution between Normal and Septic shock. (a) Stacked bar chart showing relative proportions of 22 immune cell subpopulations derived from deconvoluted analysis of pooled GEO cohort peripheral blood transcriptome data using the CIBERSORT algorithm; and (b) Box plots depicting infiltration ratios of 22 immune cell subpopulations between Normal and Septic shock conditions. [file Image6.tif]

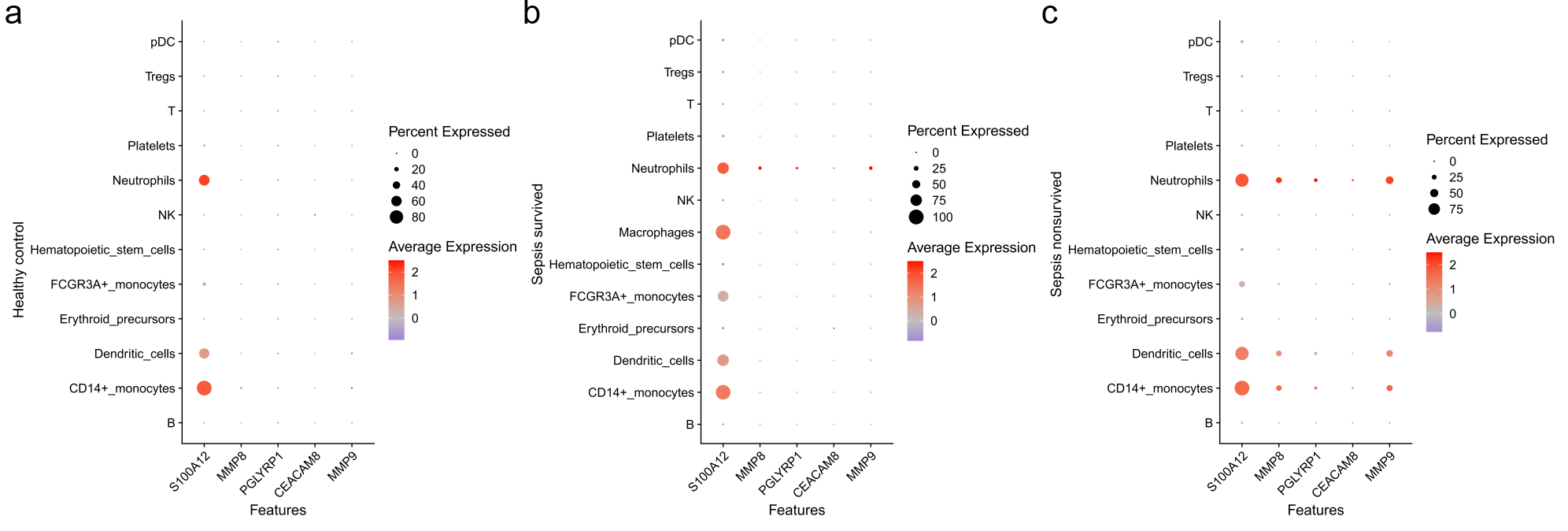

Supplement: Supplementary Figure 7 — Expression patterns of five characteristic genes in peripheral blood monocyte transcriptomes across major immune cell subpopulations and different outcome groups. (a–c) DotPlot displays the expression distribution of S100A12, MMP8, PGLYRP1, CEACAM8, and MMP9 across immune cell subpopulations, corresponding to (a) Healthy control, (b) Sepsis survived, and (c) Sepsis non-survived cohorts. [file Image7.tif]
