# Supplementary material for: Multi-cohort transcriptomics integration for building and validating a diagnostic model of peripheral blood septic shock
Source: Front Immunol. 2026 May 25;17:1768866. doi: 10.3389/fimmu.2026.1768866 (PMC13243033; doi:10.3389/fimmu.2026.1768866)
Supplement: Supplementary file 8 [file Table1.docx]

**Table S1. Sequences of siRNA used in this study.**

| Name | Sequences |
| --- | --- |
| si-h-METTL14-1 | CAACTACAATGCAGAAACA |
| si-h-METTL14-2 | GCATTGGTGCCGTGTTAAA |
| si-m-Mettl14-1 | GCAGCACCUCGGUCAUUUAdTdT |
| si-m-Mettl14-2 |  |
| si-h-YTHDF1-1 | ACGGCAGAGTCGAAACAAA |
| si-h-YTHDF1-2 | CTCCACCCATAAAGCATAA |
| si-NC | UUCUCCGAACGUGUCACGUTT |

**Table S2. Sequences of human primers used in this study.**

| Name | Sequences |
| --- | --- |
| METTL14-Forward | GTTGGAACATGGATAGCCGC |
| METTL14-Reverse | CAATGCTGTCGGCACTTTCA |
| S100A12-Forward | AGCATCTGGAGGGAATTGTCA |
| S100A12-Reverse | GCAATGGCTACCAGGGATATGAA |
| MMP8-Forward | TGCTCTTACTCCATGTGCAGA |
| MMP8-Reverse | TCCAGGTAGTCCTGAACAGTTT |
| PGLYRP1-Forward | GCCTGCCCTTACGCTATGTG |
| PGLYRP1-Reverse | CAGGAAGTTGTAGCCCACGTC |
| CEACAM8-Forward | TGCTCAGCTCACTATTGAAGC |
| CEACAM8-Reverse | CCTATAATTCGACGGTTGGCAT |
| MMP9-Forward | TGTACCGCTATGGTTACACTCG |
| MMP9-Reverse | GGCAGGGACAGTTGCTTCT |
| YTHDF1-Forward | ACCTGTCCAGCTATTACCCG |
| YTHDF1-Reverse | TGGTGAGGTATGGAATCGGAG |
| GAPDH-Forward | ATCATCCCTGCCTCTACTGG |
| GAPDH-Reverse | GTCAGGTCCACCACTGACAC |

**Table S3. Sequences of** **mouse primers used in this study.**

| Name | Sequences |
| --- | --- |
| Mettl14-Forward | GAGCTGAGAGTGCGGATAGC |
| Mettl14-Reverse | GCAGATGTATCATAGGAAGCCC |
| Gapdh-Forward | AGGTCGGTGTGAACGGATTTG |
| Gapdh-Reverse | GGGGTCGTTGATGGCAACA |
